# Clinical Characteristics of Coronavirus Disease (COVID-19) in Mexican Children and Adolescents

**DOI:** 10.3390/v14102162

**Published:** 2022-09-30

**Authors:** Alejandro Flores-Alanis, Zeus Saldaña-Ahuactzi, Israel Parra-Ortega, Pablo López-Ramírez, Marcela Salazar-García, Yolanda P. Alemán-García, Armando Cruz-Rangel, Alba Moreno-Paredes, Adrián Diaz-Rojas, Carmen Maldonado-Bernal, Jessica Quevedo-Moran, Victor M. Luna-Pineda

**Affiliations:** 1 Departamento de Microbiología y Parasitología, Facultad de Medicina, Universidad Nacional Autónoma de México, Ciudad de México 04510, Mexico; 2 Paul G. Allen School for Global Health, College of Veterinary Medicine, Washington State University, Pullman, WA 99164, USA; 3 Laboratorio Central, Hospital Infantil de México Federico Gómez, Ciudad de México 06720, Mexico; 4 Centro de Investigación en Ciencias de Información Geoespacial (CentroGeo), Ciudad de México 14240, Mexico; 5 Laboratorio de Biología del Desarrollo y Teratogénesis Experimental, Hospital Infantil de México Federico Gómez, Ciudad de México 06720, Mexico; 6 Laboratorio de Investigación en COVID-19, Hospital Infantil de México Federico Gómez, Ciudad de México 06720, Mexico; 7 Laboratorio de Bioquímica de Enfermedades Crónicas, Instituto Nacional de Medicina Genómica, Ciudad de México 14610, Mexico; 8 Unidad de Investigación en Inmunología y Proteómica, Hospital Infantil de México Federico Gómez, Ciudad de México 06720, Mexico; 9 Licenciatura en Medicina, Facultad de Medicina, Benemérita Universidad Autónoma Puebla, Puebla 72410, Mexico; 10 Licenciatura en Químico Farmacéutico Industrial, Escuela Nacional de Ciencias Biológicas, Instituto Politécnico Nacional, Ciudad de México 11340, Mexico; 11 Licenciatura en Químico Bacteriólogo Parasitólogo, Escuela Nacional de Ciencias Biológicas, Instituto Politécnico Nacional, Ciudad de México 11340, Mexico

**Keywords:** children and adolescents, COVID-19, epidemiology, comorbidities, viral load, IgG antibodies

## Abstract

Background: We analyzed the demographic, clinical, and diagnostic data of children and adolescents in Mexico, from the first case of coronavirus disease (COVID-19) to 28 February 2022. Methods: Using the open databases of the Ministry of Health and a tertiary pediatric hospital, we obtained demographic and clinical data from the beginning of the COVID-19 pandemic until 28 February 2022. In addition, quantitative reverse-transcription polymerase chain reaction outputs were used to determine the viral load, and structural protein-based serology was performed to evaluate IgG antibody levels. Results: Of the total 437,832 children and adolescents with COVID-19, 1187 died. Of these patients, 1349 were admitted to the Hospital Infantil de Mexico Federico Gómez, and 11 died. Obesity, asthma, and immunosuppression were the main comorbidities, and fever, cough, and headache were the main symptoms. In this population, many patients have a low viral load and IgG antibody levels. Conclusion: During the first 2 years of the COVID-19 pandemic in Mexico, children and adolescents had low incidence and mortality. They are a heterogeneous population, but many patients had comorbidities such as obesity, asthma, and immunosuppression; symptoms such as fever, cough, and headache; and low viral load and IgG antibodies.

## 1. Introduction

Since the coronavirus disease-2019 (COVID-19) pandemic began, 435.4 million confirmed cases and 5.94 million COVID-19-related deaths have been reported worldwide from 3 January 2020, to 28 February 2022, according to the World Health Organization (https://www.who.int/es [accessed on 28 February 2022]). Mexico has experienced 5.5 million confirmed cases and 318,149 COVID-19-related deaths; additionally, it has experienced five waves of the COVID-19 pandemic, with the first wave reaching its peak in July 2020; the second wave, December 2020; the third wave, August 2021; the fourth wave, January 2022; and the fifth wave is ongoing (https://datos.covid-19.conacyt.mx/ [accessed on 28 April 2022]). In adults, pre-existing comorbidities are significant risk factors for severe acute respiratory syndrome coronavirus 2 (SARS-CoV-2) [1]. Viral load is also recognized as a strong determinant of transmission risk [2]. Children mostly contract mild COVID-19, but the extent of pediatric COVID-19 course differs between geographical regions and distinct pandemic waves. Children may play a major role in community-based viral transmission. Available data suggest that children may have more upper respiratory tract involvement, including nasopharyngeal carriage, than lower respiratory tract involvement [3].

Some children develop severe complications such as multisystem inflammatory syndrome (MIS-C) [4,5]. Interestingly, in children with cancer and allergies, the rates of mortality and complications of COVID-19 did not increase, but questions remain regarding other childhood comorbidities, viral load, and COVID-19-associated outcomes [6,7]. During the initial phase of the COVID-19 outbreak in China, less than 1% of the cases occurred in children aged <10 years [8,9]. Similarly, during the first wave in Italy, only 1% of patients with COVID-19 were children; 21% of the infected children were asymptomatic, 58% had mild disease, and 11% were hospitalized, but none died [10].

However, data regarding the demographics and clinical features of COVID-19 in children from Mexico are limited. Thus, in this study, we aimed to present the epidemiology of COVID-19 in children and adolescents during the first four waves of the pandemic in Mexico. We focused on patients from a tertiary pediatric care hospital in Mexico City to investigate the associations between demographic, clinical, and diagnostic information to identify possible COVID-19 profiles. The collection of COVID-19 profiles helps in understanding COVID-19 outcomes in children and adolescents.

## 2. Materials and Methods

### 2.1. Study Design

**Figure 1:**
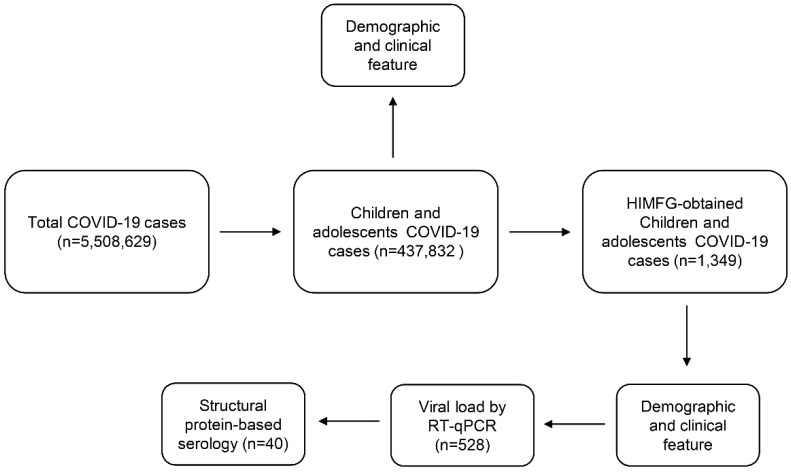
Flowchart of the study of children and adolescents in Mexico. From the beginning of the COVID-19 pandemic until 28 February 2022, 5,508,629 cases were diagnosed, of which 437,832 were children and adolescents. In the HIMFG, 1349 children and adolescents were admitted, where 528 were diagnosed by RT-qPCR. CTs were obtained of RT-qPCR outputs, and viral load was determined as low, moderate, or high. Structural protein-based serology was performed on a small group of 40 children and adolescents.

The study included children and adolescents with COVID-19 from 29 February 2020, to 28 February 2022. The children included were neonates and babies (<2 years old), toddlers (2–4 years old), and schoolchildren (5–9 years old). The adolescent group (10–19 years old) was subclassified into early adolescents (10–14 years old) and late adolescents (15–19 years old). Demographic and clinical data were collected from the Mexican Government and Hospital Infantil de Mexico Federico Gómez (HIMFG) databases. In addition, we performed COVID-19 structural protein-based serology by enzyme-linked immunosorbent assays (ELISA) on a group of 40 children and adolescents with COVID-19, which were confirmed by reverse-transcription (RT)-quantitative polymerase chain reaction (qPCR) and clinical data (Figure 1).

### 2.2. Data Sources

#### 2.2.1. National Data: Mexico

Data were obtained from the open database of the Ministry of Health, which publishes daily updates on all patients with COVID-19 (https://datos.covid-19.conacyt.mx/ [accessed on 28 April 2022]). It includes suspected cases (due to the clinical picture or an epidemiological association) and confirmed cases by three processes: test confirmed by an authorized laboratory, epidemiological association with a case confirmed by a laboratory test, and cases confirmed by an opinion committee (only occurs in the case of deaths). The inclusion criteria were as follows: patients aged ≤19 years with confirmed records of COVID-19 diagnosis. The patients were subsequently grouped according to residence, epidemiological week, and age group, whereas clinical features were patient status and comorbidities. The temporal incidence of confirmed COVID-19 and deaths from 28 February 2020, to 28 February 2022, is presented in a graph with the number of cases per week.

The incidence of COVID-19 in Mexico per 100,000 inhabitants was used to generate the geographical distribution across the 32 states of the Mexican territory. The total population for each state was obtained from the most recent records of the Mexican Census of Population and Housing 2020, carried out by Instituto Nacional de Estadística, Geografía e Informática (https://www.inegi.org.mx/ [accessed on 28 July 2022]). The age groups of children and adolescents were used to create a heat map. Information was obtained, and confirmed COVID-19 cases were identified in relation to the number of hospitalized patients and ambulatories; the number of deaths, intubated, and pediatric cases, in an intensive care unit (ICU); and pediatric patients with comorbidities such as diabetes, chronic obstructive pulmonary disease (COPD), asthma, immunosuppression, hypertension, cardiovascular disorder, obesity, chronic kidney disease, smoking, and other comorbidities. Immunosuppression was defined as patients who received immunosuppressors and/or glucocorticoids for autoimmune diseases, chemotherapy, and hemato-oncological diseases, including immunosuppression therapy (transplant procedures), but excluding HIV-positive cases. Other comorbidities included hepatic insufficiency, sepsis, and cerebrovascular events.

#### 2.2.2. Regional Data: HIMFG

The HIMFG COVID-19 database was downloaded from the Sistema Nacional de Vigilancia Epidemiológica (https://www.sinave.gob.mx/ [accessed on 23 March 2022]) and the COVID-19 test database from the Central Laboratory-HIMFG. Demographic data included sex and age, and clinical features were patient status, comorbidities, signs, and symptoms. The type of SARS-CoV-2 infection was classified as asymptomatic in patients without any COVID-19 symptoms and attended ambulatorily; mild COVID-19, patients with signs and symptoms but with ambulatory management; moderate COVID-19, patients with mild illness but with hospital management; and severe COVID-19, patients who were admitted to the ICU for COVID-19, were intubated, or died [11]. The HIMFG is a tertiary-care pediatric hospital that admits a large population of children and adolescents for surgery, metabolic disease, organ transplants, and chemotherapy. However, during the COVID-19 pandemic, it has been a reference hospital to attend this disease. Therefore, all patients admitted to the HIMFG were tested for SARS-CoV-2 as part of the hospital’s protocol. This population was considered to have asymptomatic SARS-CoV-2 infection when they lacked signs and symptoms of COVID-19 during their hospital stay or if they died for an underlying disease.

The datasets were obtained to only keep patients who tested positive for SARS-CoV-2 by a COVID-19 Ag Rapid Test Panbio™ and RT-qPCR. The cycle threshold (Ct) values were recovered from RT-qPCR outputs, and these values were considered for the determination of viral load as described by Maltezou et al. [12]. In addition, the Ct values were used to determine the number of viruses for each viral load. It was calculated using the following equation, y = −3.2733 x + 38.59, as described in a previous study [13]. Low viral load was defined as Ct value 30–38 (4.2 × 10^2^–1.5 copies number/mL), moderate as Ct value 25–29 (1.4 × 10^4^–8.5 × 10^2^ copies number/mL), and high as Ct value <24 (<2.8 × 10^4^ copies number/mL).

Structural protein-based serology was performed using indirect ELISA with recombinant structural proteins of SARS-CoV-2 (RBD-S, N, E, and M proteins), as antigens that were obtained in a previous study [11]. During this study, a biobank was generated in HIMFG during the COVID-19 pandemic for children and adolescents whose parents accepted and signed the informed consent form. Only a small group followed the protocol and fulfilled the inclusion criteria. The criteria were as follows: (1) blood sampling after 30 days without signs and symptoms of COVID-19 (convalescent), (2) positive RT-qPCR test with Ct value ≤38, and (3) inclusion in the SIVAVE database (demographic and clinical data). Briefly, antigen coating was performed on 96-well plate with 100 µL per well of each recombinant protein at a final concentration of 0.1 µg protein/mL in sensitization buffer (50 mM Na_2_CO_3_/Na_2_CO_3_H, pH 9.6). The coated plate was incubated for 1 h at 37 °C, washed, and then blocked for 30 min with 200 µL/well of 5% skim milk in phosphate-buffered saline (PBS)–Tween 20 (0.05%). The initial wash steps (coating and blocking) were performed three times per 5 min with PBS–Tween 20 (0.05%) and agitated at 70 rpm. The antibodies were washed five times for 7 min with the same agitation. The sera of each participant were diluted at 1:50 in PBS (pH 7.2) and were added to 100 µL/well by duplicate. These were incubated for 1 h and then washed. The anti-human IgG antibodies (1:2000 dilution, Sigma-Aldrich, MO, USA) coupled to horseradish peroxidase were added to 100 µL/well and incubated for 1 h, and then washed. The enzymatic reaction was conducted using o-Phenylenediamine Dihydrochloride (Sigma-Aldrich), and the reaction was stopped with H_2_SO_4_ 2N. Optical density was measured at 492 nm using a microplate reader. The negative threshold value or cutoff was calculated by the Student t-distribution with 99.9% using the mean ($\bar{X}$) and its standard deviation (SD) values and confidence level (*t* = 1 − α) from 10 sera of pre-pandemic patients. The equation used was $Cutoff=\bar{X}+\mathrm{SD}t\sqrt{1+\left(1/n\right)}$ [14]. The IgG antibody values were also calculated using *X* and SD values from all sera with a value above the negative cutoff. From the negative threshold value, 1 SD was considered low level, 2 SD as moderate level, and 3 SD as a high level of IgG antibodies. The sensitivity analysis was performed using a commercial test according to the manufacturer (sensitivity 100%, specificity 99.8%), and the results were compared with the in-house serology test validation.

### 2.3. Statistical Analysis and Graphs

**Figure 2:**
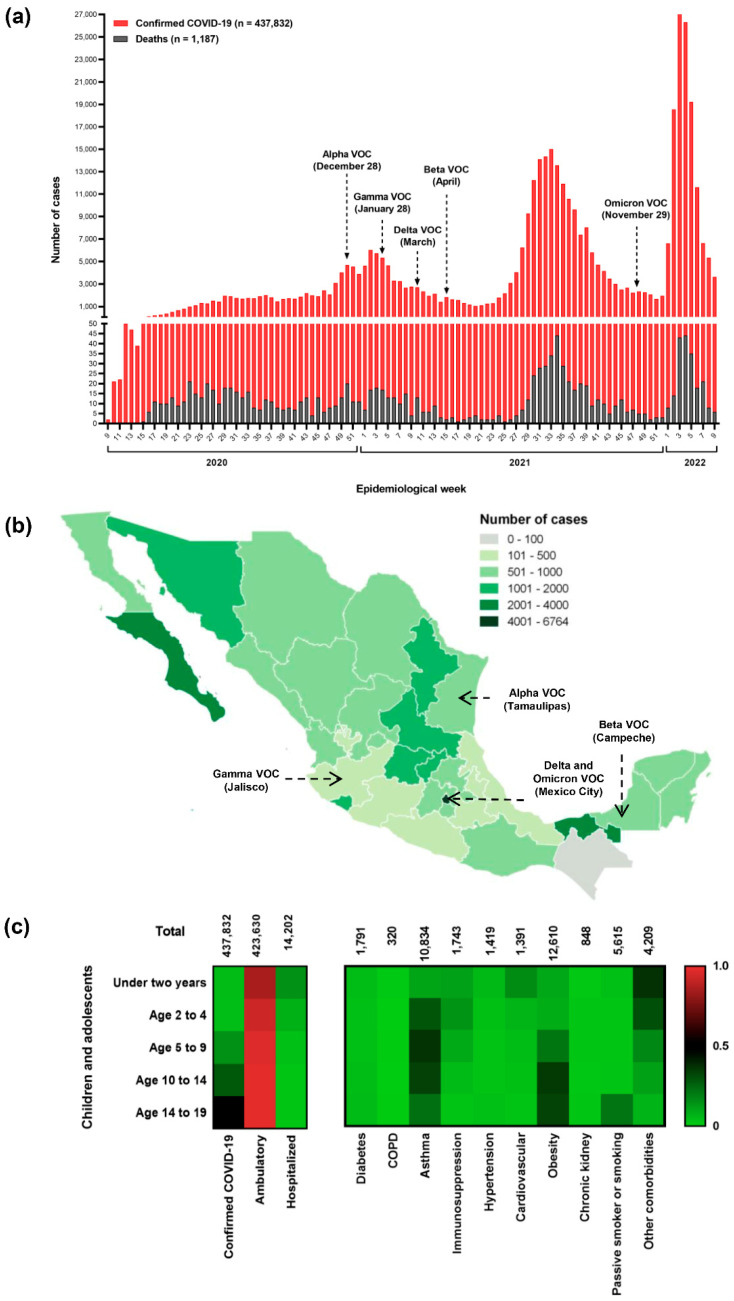
Snapshot of the COVID-19 pandemic in children and adolescents in Mexico: (**a**) temporal incidence of confirmed cases and deaths from COVID-19 from 29 February 2020, to 28 February 2022; (**b**) geographical distribution of the number of cases per 100,000 habitats across the 32 states of Mexico; (**c**) heat map by age group of the clinical presentations of the population analyzed.

Categorical variables were expressed as absolute (n) and relative (%) values with 95% confidence intervals (CIs), and the Chi-square test was used to evaluate significant differences. Multivariable logistic regression models were performed to evaluate the association between patient care (ambulatory or hospitalized), viral load (low, moderate, or high), and risk factors such as age, sex, SARS-CoV-2 infection, comorbidities, and signs/symptoms. Correlation analysis between viral load and antibodies levels with clinical and demographic variables was performed using Cramer’s V coefficient and Fisher’s exact test via contingency tables; when not possible, the Monte Carlo method was used [14]. In all tests, *p* < 0.05 was considered significant. All statistical analyses were performed using the stats v4.1.3 and DescTools v.0.99.45 R packages from RStudio v3.2.2 (https://rstudio.com/ (accessed on 28 April 2022)). The graph and heat map (Figure 2) was made using GraphPad Prism version 9.0.0 for Windows (GraphPad Software, San Diego, CA, USA; https://www.graphpad.com (accessed on 28 April 2022)). The Mexican heat state map was made using QGIS v3.22.7 software (https://www.qgis.org/en/site/ (accessed on 28 April 2022)).

## 3. Results

### 3.1. National Outlook of the Pediatric and Adolescent Population

In Mexico, 5,508,629 COVID-19 cases have been reported up to 28 February 2022; of them, 437,832 (7.95%) were in children and adolescents, with a fatality rate of 0.27% (*n* = 1187). The first case of COVID-19 in this age group was a 19-year-old female teen from Chiapas. Since then, the number of cases has increased over time (Figure 2a). Mexico has endured four waves of the COVID-19 pandemic and is now experiencing a fifth wave. However, in this study, we analyzed only four waves, from the first case of COVID-19 in Mexico to 28 February 2022. The peak of the second wave coincided with the detection of the variant of concern (VOC) alpha in Mexico. Meanwhile, the cases associated with the gamma, delta, and beta VOCs preceded the third wave, and omicron VOC was detected in the fourth wave. In total, 1034 deaths were reported, surpassing the highest number during the first wave (*n* = 301) with a 1% mortality rate (Figure 2a). The states with the highest number of cases per 100,000 habitants were Mexico City (*n* = 6764), Baja California Sur (*n* = 3449), and Tabasco (*n* = 2208). By contrast, Chiapas had the lowest number of cases (*n* = 71) (Figure 2b).

Overall, the number of cases increased according to age group. The highest number of confirmed cases was recorded in the group aged 14–19 years (*n* = 213,540 [48.77%]), and the lowest was in groups aged 2–4 (*n* = 17,477 [3.99%]) and <2 (*n* = 19,515 [4.46%]) years. Most of the patients were ambulatories (*n* = 423,630 [96.76%]). Of the hospitalized patients (*n* = 14,202 [3.24%]), the majority were adolescents aged 14–19 years (*n* = 4822 [33.95%]), followed by neonates and babies aged <2 years (*n* = 2893 [20.37%]) (Figure 2c). Nine comorbidities were detected among the populations analyzed. The principal comorbidities were obesity (*n* = 12,610 [2.88%]) and asthma (*n* = 10,834 [2.47%]), and both were more frequent in the group aged 14–19 years with 7861 cases (62.33%) and 5393 cases (49.77%), respectively. When children and adolescents were compared between age groups, cardiovascular disease was mainly detected in neonates and babies aged <2 years and immunosuppression in preschoolers aged 2–4 years (Figure 2c).

### 3.2. Perspectives of a Tertiary-Care Pediatric Hospital (HIMFG)

HIMFG is a tertiary-care hospital in Mexico City and cares for children and adolescents with COVID-19. Throughout the pandemic, it has treated 1349 (0.61%) children and adolescents of the total confirmed COVID-19 cases (*n* = 437,832) in Mexico up to 28 February 2022; of them, only 11 patients died, with a fatality rate of 0.81% (Table 1).

The prevalence of COVID-19 was significantly higher in males (54.4%) than in females (45.6%) (*p* < 0.05). The median age was 12.92 ± 5.3 years, and most patients were between 10–14 (23.3%) and 5–9 (22.2%) years old. RT-qPCR and rapid antigen tests were used to diagnose COVID-19; nevertheless, only 39.1% (*n* = 528) of the patients were diagnosed by molecular test. Of them, 48.7% presented low, 38.4% high, and 12.9% moderate viral loads. Cases of SARS-CoV-2 infection were mild in 41.3% (*p* < 0.001), asymptomatic in 31.9%, moderate in 23.9%, and severe in 2.9% of the patients. Of the 10 comorbidities detected in the population analyzed, three were the most frequent, namely, immunosuppression (9.3%), cardiovascular diseases (no hypertension) (3.6%), and obesity (3.1%). Moreover, fever (43.9%), cough (31.1%), headache (18.1%), irritability (17%), and rhinorrhea (15%) were the most frequent signs/symptoms (Table 1).

A multivariable logistic regression test showed that comorbidities such as immunosuppression (OR 2.85, 95% CI 1.88–4.33, *p* < 0.001) and cardiovascular disease (no hypertension) (OR 2.14, 95% CI 1.15–4.02, *p* < 0.05) and signs/symptoms such as abdominal pain (OR 1.52, 95% CI 1.02–2.25, *p* < 0.05), diarrhea (OR 1.47, 95% CI 1.00–2.15, *p* < 0.05), and anosmia (OR 2.95, 95% CI 1.11–8.25, *p* < 0.05) were associated with hospitalization. By contrast, variables such as age (2–4 years [OR 2.08, 95% CI 1.39–3.11, *p* < 0.001] and 10–14 years [OR 1.51, 95% CI 1.05–2.16, *p* < 0.05]), signs/symptoms such as rhinorrhea (OR 1.46, 95% CI 1.01–2.13, *p* < 0.05), and dysgeusia (OR 4.61, 95% CI 1.55–14.82, *p* < 0.01) were associated with ambulatory.

#### 3.2.1. Features of Ambulatory and Hospitalized Patients Who Attended HIMFG

We investigated possible COVID-19 profiles between the ambulatory group and the hospitalized group. All ambulatory patients (*n* = 846) had mild and asymptomatic disease; among these patients, most did not present with comorbidities (*n* = 696; 82.27%), 15.13% (*n* = 128) presented with one comorbidity, and 2.6% (*n* = 22) presented with 2–4 comorbidities. Among the hospitalized group (*n* = 503), 323 (64.21%) and 39 (7.75%) patients had moderate and severe diseases, respectively. Interestingly, 141 (28.03%) patients in the hospitalized group were asymptomatic. Similar to the ambulatory group, most patients did not present with comorbidities (*n* = 343, 68.19%), 23.26% (*n* = 117) presented with one comorbidity, and 8.55% (*n* = 43) presented with 2–5 comorbidities. Nevertheless, the incidence of immunosuppression was higher in the hospitalized group than in the ambulatory group (*p* < 0.05). By contrast, the incidence of asthma was higher in the ambulatory group than in the hospitalized group (*p* < 0.05) (Table 1).

#### 3.2.2. Analysis of Viral Load and Serology from Patients Diagnosed through RT-PCR

The comparison between sex and viral loads only showed a significant difference in the group with high viral load, and males were the most affected (57.1%, *p* < 0.05) (Table 2).

By age group, and taking children aged <2 years as the reference, significant differences were only observed in children aged 5–14 years (*p* < 0.001). Similar results were observed in patients with low viral load, but no significant differences were observed in those with moderate and high viral loads (Table 2). Patients were primarily ambulatories (59.3%, *p* < 0.05), and significant differences were detected in patients with low and high viral loads (*p* < 0.05) (Table 2). Moreover, a low viral load was observed in mild (38.1%) and asymptomatic cases (32.7%), with a significant difference when compared with moderate (23.3%, *p* < 0.05) and severe (5.8%, *p* < 0.001) cases. Similar results were observed in moderate and severe viral loads (Table 2).

Using a multivariable logistic regression analysis, we evaluated if demographic and clinical features were associated with viral load. Age (5–9 years) was associated with low viral load (OR 2.11, 95% CI 1.16–3.88, *p* < 0.05); headache (OR 2.50, 95% CI 1.04–5.91, *p* < 0.05) and dysgeusia (OR 9.47, 95% CI 1.10–104, *p* < 0.05) were associated with moderate viral load; whereas chills were associated with high viral load (OR 2.11, 95% CI 1.07–4.21, *p* < 0.05).

**Figure 3:**
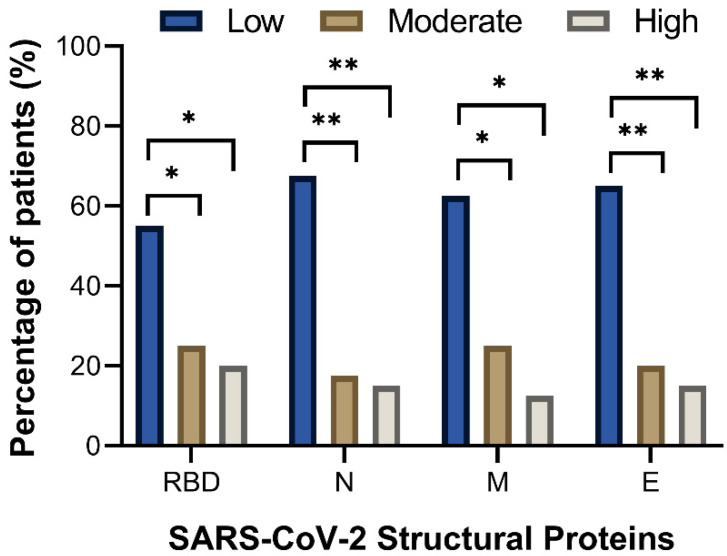
Levels of IgG antibodies from Mexican children and adolescents with COVID-19. IgG antibodies were determined by ELISA using SARS-CoV-2 RBD, N, E, and M proteins and samples from 40 patients in convalescence who had positive RT-qPCR test (Ct value), demographic, and clinical data. The negative threshold value was an optical density at 492 nm (OD_492_) of 0.006. A low level of IgG antibodies was defined as an OD_492_ of >0.06 to 0.13 for RBD protein, and an OD_492_ of >0.06 to 0.17 for N, M, and E proteins. A moderate level of IgG antibodies was defined as an OD_492_ of >0.13 to 0.45 for RBD protein, OD_492_ > 0.17 to 0.46 for N and M proteins, and OD_492_ > 0.17 to 0.43 for E protein. Finally, a high level of IgG antibodies was defined as an OD_492_ of >0.45 for RBD protein, OD_492_ >0.46 for N and M proteins, and OD_492_ > 0.43 for E protein. Significance was considered * *p* < 0.05 for RBD and M proteins, and ** *p* < 0.001 for N and E proteins.

We performed a serological analysis of 40 patients with convalescent COVID-19 to detect IgG antibody levels against four SARS-CoV-2 antigens. The negative threshold value was an optical density at 492 nm (OD492) of 0.006. A low level of IgG antibodies was defined as an OD492 of >0.06 to 0.13 for RBD protein and an OD492 of >0.06 to 0.17 for N, M, and E proteins. A moderate level of IgG antibodies was defined as an OD492 of >0.13 to 0.45 for RBD protein, OD492 >0.17 to 0.46 for N and M proteins, and OD492 >0.17 to 0.43 for E protein. Finally, a high level of IgG antibodies was defined as an OD492 of >0.45 for RBD protein, OD492 >0.46 for N and M proteins, and OD492 >0.43 for E protein. Most patients had low levels of IgG antibodies for the four antigens tested: RBD protein (*n* = 22, 55%), N protein (*n* = 27, 67.5%), M protein (*n* = 25, 55%), and E protein (*n* = 26, 65%). Significant differences were found when compared with patients with moderate and high IgG antibody levels (Figure 3). However, no significant associations were observed when we compared the levels of IgG antibodies of each antigen against viral load, patient care, SARS-CoV-2 infection, and sex (Fisher’s exact test, *p* > 0.05).

#### 3.2.3. Evaluation of Deceased Patients Who Attended HIMFG

All patients who died (*n* = 11) were mainly boys aged >10 years and from the hospitalized group. This group was identified on the first wave and the beginning of the second wave of the COVID-19 pandemic (May to December 2020). Of these 11 patients, 8 (63.63%) had a severe infection and 3 (27.27%) were asymptomatic. Seven (63.63%) had a low viral load, one (9.9%) had a high viral load, and three (27.27%) were diagnosed by antigen test. Four comorbidities were detected, namely, immunosuppression (*n* = 5, 45.45%), cardiovascular disease (no hypertension) (*n* = 2, 18.18%), diabetes mellitus (*n* = 1, 9.9%) and chronic kidney disease (*n* = 1, 9.9%); and two patients had no comorbidities. Nine (81.81%) were intubated. Interestingly, two patients with cardiovascular diseases were neonates with asymptomatic COVID-19. Fever (*n* = 5, 45.45%), cough and dyspnea (*n* = 4, 36.36% each) were the more frequent sign/symptoms. A 17-year-old patient with a more severe clinical history, who was intubated, was the only patient with two comorbidities (immunosuppression and chronic kidney disease) and presented with 11 signs/symptoms (i.e., fever, cough, dyspnea, diarrhea, thoracic pain, chills, arthralgia, polypnea, vomiting, abdominal pain, cyanosis, and anosmia).

## 4. Discussion

Our national epidemiology data from children and adolescents with COVID-19 in Mexico for the 2 years of the pandemic, showed a clear increase in the incidence with age and a low mortality rate. Adolescents showed the highest incidence, similar to a previous report in Mexico [15]. In the HIMFG, COVID-19 cases were slightly higher in boys than in girls, similar to other epidemiological studies [16,17]. Nevertheless, we found a slight increase in the proportion of severe cases in neonates, toddlers aged >2 years, and early adolescents, compared with the rest of the age groups. Other epidemiological studies from China, India, and the U.S.A. have reported that young children, particularly infants, were at risk for COVID-19 [16,18,19].

Most SARS-CoV-2 infections in children are non-severe, and children are less likely to develop severe diseases than adults [19]. Moreover, <3% of children have developed severe or critical disease [20,21,22]. Similarly, in HIMFG, fewer children developed severe illness, and most symptomatic children presented with mild and moderate symptoms. Of the children and adolescents with symptoms, fever was present at any time during the illness. Other common signs and symptoms included cough and headache. Other retrospective studies from the U.S.A., China, and India have reported similar signs and symptoms [16,23,24]. Children may have had more upper respiratory tract involvement than lower respiratory tract involvement [25]. Although most children and adolescents with COVID-19 admitted to the HIMFG experienced mild illness, some developed a severe disease that led to hospitalization, and some of them died. A large group of children was diagnosed with asymptomatic COVID-19. This group included patients who tested for SARS-CoV-2 during their appointment for other pre-existing diseases as part of the hospital protocol. The asymptomatic population reflected the sub-estimation of the actual number of COVID-19 cases in Mexico and other regions.

In Mexico, SARS-CoV-2 VOCs were introduced mainly from the U.S.A. and Europe, and they were responsible for the four waves described in this study [26]. In 2020, the most prevalent SARS-CoV-2 lineages were B.1 and B.1.222. These variants were likely the cause of the high mortality rate in the first wave [26]. Mexico City had the highest number of cases and deaths, and the HIMFG is a tertiary-care pediatric hospital located in Mexico City. Children and adolescents who died of COVID-19 in the HIMFG were identified between May and December 2020 (first and second waves). Nevertheless, in the first wave of the pandemic, the mortality rate in this population was lower than that in other studies from a different tertiary hospital in Mexico [27]. In addition, other studies from Brazil and Indonesia have reported higher mortality rates, with 15% and 40%, respectively [28,29]. This difference might be due to the significantly higher number of patients in this study, which includes hospitalized and ambulatory patients from the four waves of the pandemic. For example, a study from China with a high number of patients from the first three pandemic waves showed a similar mortality rate (0.9%) [30]. No deaths were reported in a study from a tertiary-care setting in Thailand with 1019 patients from three waves of the pandemic [17].

This population often present with one or more pre-existing underlying chronic diseases that will affect their prognoses and mortality. We observed a tendency to increases in the number of COVID-19 cases with obesity for all age groups, similar to those in other studies [28,31]. The population with obesity had a 3.1 times higher risk of being hospitalized and 1.4 times higher risk of having severe illness during hospitalization [31]. Similarly, a recent study from Mexico found an association between obesity and a worse COVID-19 prognosis [15]. It must be emphasized that Mexico has the highest prevalence of overweight children and adolescents globally; therefore, it is essential to pay attention to these patients with COVID-19 [32]. Asthma, a comorbidity with high incidence in Mexico, is also associated with poor prognosis and mortality when compared with other patients with COVID-19 [33], in whom immunosuppression and diabetes were other comorbidities identified with high incidence. Patients with immunosuppression and malignancy and recipients of solid-organ transplantation have been associated with an increased risk of severe COVID-19 and death, in addition to type 1 and type 2 diabetes [34,35,36]. In the HIMFG, immunosuppression, cardiovascular disease, obesity, and asthma had the highest incidence in patients with COVID-19. In Mexico, obesity in children is very common and strongly related to COVID-19 and severe illness [15]. In this population, underlying conditions such as asthma and obesity were also associated with severe COVID-19 [11,37]. A systematic review of critically unwell children and the association with underlying comorbidities showed that those with pre-existing cardiac diseases were critically unwell [38]. Two neonates admitted to the HIMFG with cardiovascular diseases were critically unwell and died. Other children and adolescents with pre-existing immunosuppression, diabetes, and chronic kidney disease died during the first wave of the COVID-19 pandemic. On the contrary, three patients with asymptomatic COVID-19 arrived at the hospital for cardiovascular diseases and one for surgery, and they died unfortunately. They were tested for SARS-CoV-2 as part of the hospital protocol. These results showed the great response of our hospital during the 2 years of the COVID-19 pandemic.

The increased detection of asymptomatic COVID-19 in children and adolescents has a beneficial effect on SARS-CoV-2 spread and subsequent decrease in COVID-19 mortality [39]. The HIMFG used RT-qPCR and rapid antigen tests to diagnose COVID-19. Both tests show similar trends in the incidence of COVID-19 [40]. The HIMFG is a tertiary care pediatric hospital that attends to a large population of hospitalized children and adolescents for surgery, metabolic disease attention, and treating patients with leukemia who require outpatient chemotherapy. All patients are tested for COVID-19 as part of the hospital protocol. A rapid test was used to diagnose patients with symptomatic and asymptomatic COVID-19. RNA extraction from swab samples and RT-qPCR test were performed for COVID-19 confirmation only if the rapid test was negative. This explains why many outpatients and hospitalized patients were diagnosed by rapid testing.

Many patients diagnosed in HIMFG did not have any symptoms of infection, which correlates with the results of a meta-analysis of asymptomatic COVID-19 [41]. Despite the low risk, the asymptomatic group with a high SARS-CoV-2 viral load may play a significant role in community-based viral transmission. On the contrary, patients aged < 5 years were characterized by a higher SARS-CoV-2 viral load in the nasopharynx than the adolescent population [42]. However, in this study, we did not find a significant difference in SARS-CoV-2 viral load in all age groups. Additionally, the high SARS-CoV-2 viral load was an independent predictor of symptomatology, disease severity, and mortality. Therefore, serological testing of children and adolescents with asymptomatic disease is essential to evaluate the infection and spread of SARS-CoV-2 [43]. We used the ELISA and we evaluated the antibody response against the SARS-CoV-2 structural protein in a small group of 40 patients. This group had clinical follow-ups, and patients were considered convalescent. A study showed that comorbidities in this population play an essential role in the antibody response against SARS-CoV-2 infection [11]. However, we did not identify a tendency in this population and only found a high incidence of low antibody levels. On the contrary, the COVID-19 vaccine for children ages 5–11 years was authorized by the FDA in the U.S.A. at the beginning of November 2021. However, in Mexico, vaccination was approved only for children with comorbidities in November 2021 after a court ruled for vaccination in this group. The vaccination registration for all those aged >18 years was in July 2021, whereas for those aged 12–17 years with chronic diseases, it was in October 2021. In addition, the vaccination of children and adolescents from 15 to 17 years was in November 2021, and for children from 12 to 14 years in July 2021. Finally, the COVID-19 vaccine for girls and boys aged 5–11 years was in March 2022 (https://vacunacovid.gob.mx/ [accessed on 8 September 2022]). Our population included neonates and babies (<2 years old), toddlers (2–4 years old), schoolchildren (5–9 years old), early adolescents (10–14 years old), and late adolescents (15–19 years old). We analyzed this population from the first case of COVID-19 to 28 February 2022, when only the adolescent group (≥12 years old) was likely to be vaccinated against COVID-19. Regrettably, when we obtained the open databases, information about the vaccinated population was unavailable.

## 5. Strengths and Weakness

Our findings should be considered in light of some limitations. First, the study was designed to investigate the associations between demographic, clinical, and diagnostic information to identify possible COVID-19 profiles, and the estimates reported herein do not reflect any causal effects. Second, our data were collected from a single tertiary pediatric care hospital in Mexico. Third, we did not analyze the sociodemographic data regarding ethnicity, education, and parents’ income, which were associated with COVID-19 outcomes in an early study from Mexico [15]. Fourth, a selection bias in the patients reported resulted from the study being conducted in a tertiary pediatric care hospital for managing patients with COVID-19, which is also a referral hospital for patients with different comorbidities. Despite these limitations, this study revealed a low mortality rate in pediatric patients with COVID-19. Additionally, this study has several strengths. Data from a relevant number of Mexican patients were analyzed.

## 6. Conclusions

During the 2 years of the COVID-19 pandemic in Mexico, a low number of cases and mortality was recorded in children and adolescents. Obesity, asthma, immunosuppression, and cardiovascular disease were the main comorbidities. Mexico City was the state with the highest number of cases, where the HIMFG had successfully attended to children and adolescents with COVID-19, with only 11 dying. Moreover, patients who attended HIMFG had comorbidities similar to national data. They are a heterogeneous population, but comorbidities such as obesity, asthma, and immunosuppression, symptoms such as fever, cough, and headache, and low viral load and IgG antibodies, were the main clinical features in this population. These findings must be considered to understand COVID-19 outcomes in this population.

## Figures and Tables

**Table 1:** Demographic and clinical characteristics of the population treated at the Hospital Infantil de Mexico “Federico Gómez”.

Variable	Total *n* (%, 95% CI)	Ambulatory *n* (%, 95% CI)	Hospitalized *n* (%, 95% CI)	*p* Value
**Patient care**	1349 (100)	846 (62.7,60.1–65.2)	503 (37.3, 34.7–39.9)	<0.001
**Sex**				
Female	612 (45.4, 42.7–48.0)	383 (62.6, 58.7–66.3)	229 (37.4, 33.7–41.3)	<0.001
Male	737 (54.6, 52.0–57.3)	463 (62.8, 59.3–66.2)	274 (37.2, 33.8–40.7)	<0.001
**Age (years)**				
<2	265 (19.6, 17.6–21.8)	157 (59.2, 53.2–49.9)	108 (40.8, 35.0–46.8)	<0.01
2–4	219 (16.2, 14.4–18.3)	156 (71.2, 64.9–76.8)	63 (28.8, 23.2–35.1)	<0.001
5–9	299 (22.2, 20.0–24.4)	189 (63.2, 57.6–68.5)	110 (36.8, 31.5–42.4)	<0.001
10–14	315 (23.3, 21.2–25.7)	203 (64.4, 59.0–69.5)	112 (35.6, 30.5–41.0)	<0.001
15–19	251 (18.6, 16.6–20.8)	141 (56.2, 50.0–62.2)	110 (43.8, 37.8–50.0)	ns
**Diagnostic**				
RT-qPCR	528 (39.1, 36.6–41.8)	241 (45.6, 41.4–50.0)	287 (54.3, 50.1–58.5)	<0.05
Rapid antigen test	821 (60.9, 58.2–63.4)	371 (45.2, 41.8–48.6)	450 (54.8, 51.4–58.2)	<0.05
**Viral load**				
Low	257 (19, 17.0–21.2)	152 (59.1, 53.0–65.0)	105 (40.9, 35.0–46.9)	<0.05
Moderate	68 (5, 4.0–6.3)	38 (55.9, 44.1–67.0)	30 (44.1, 32.9–55.9)	ns
High	203 (15, 13.2–17.0)	123 (60.6, 53.7–67.0)	80 (39.4, 32.9–46.3)	<0.05
Non-determinate	821 (61, 58.2–63.4)	533 (64.9, 61.6–68.1)	288 (35.1, 31.9–38.4)	<0.001
**SARS-CoV-2 infection**				
Asymptomatic	430 (31.9, 29.4–34.4)	289 (67.2, 62.6–71.5)	141 (32.8, 28.5–37.4)	<0.001
Mild	557 (41.3, 38.7–43.9)	557 (100)	0	ND
Moderate	323 (23.9, 21.7–26.3)	0	323 (100)	ND
Severe	39 (2.9, 2.1–3.9)	0	39 (100)	ND
**Comorbidities**				
Immunosuppression	126 (9.3, 7.9–11.0)	50 (39.7, 31.6–48.4)	76 (60.3, 51.6–68.4)	<0.05
Cardiovascular disease (no hypertension)	48 (3.6, 2.7–4.7)	21 (43.7, 30.7–57.7)	27 (56.3, 42.3–69.3)	ns
Obesity	42 (3.1, 2.3–4.2)	21 (50.0, 35.5–64.5)	21 (50.0, 35.5–64.5)	ns
Asthma	36 (2.7, 1.9–3.7)	24 (66.7, 50.3–79.8)	12 (33.3, 20.2–49.7)	<0.05
Chronic kidney disease	36 (2.7, 1.9–3.7)	19 (52.8, 37.0–68.0)	17 (47.2, 32.0–63.0)	ns
Chronic hypertension	16 (1.2, 0.7–1.9)	8 (50.0, 28.0–63.0)	8 (50.0, 28.0–63.0)	ns
Diabetes mellitus	9 (0.7, 0.3–1.2)	4 (44.4, 18.9–73.3)	5 (55.6, 26.7–81.1)	ns
Passive smoker or smoking	9 (0.7, 0.3–1.2)	4 (44.4, 18.9–73.3)	5 (55.6, 26.7–81.1)	ns
HIV	3 (0.2, 0.07–0.65)	2 (67.0, 20.8–93.8)	1 (33.0, 6.15–72.2)	ND
COPD	2 (0.1, 0.04–0.54)	1 (50.0, 9.45–90.5)	1 (50.0, 9.45–90.5)	ND
Other diseases	63 (4.7, 3.7–5.9)	24 (38.1, 27.1–50.4)	39 (61.9, 49.5–72.9)	ns
**Signs/Symptoms**				
Fever	593 (43.9, 41.3–46.6)	367 (61.9, 57.9–65.7)	226 (38.1, 34.3–42.1)	<0.001
Cough	420 (31.1, 28.7–33.6)	270 (64.3, 59.6–68.7)	150 (35.7, 31.3–40.4)	<0.001
Headache	244 (18.1, 16.1–20.2)	154 (63.1, 56.9–68.9)	90 (36.9, 31.1–43.1)	<0.001
Irritability	230 (17, 15.1–19.1)	136 (59.1, 52.7–65.3)	94 (40.9, 34.7–47.3)	<0.001
Rhinorrhea	203 (15, 13.2–17.0)	136 (67.0, 60.3–73.1)	67 (33.0, 26.9–39.7)	<0.001
Abdominal pain	179 (13.3, 11.6–15.2)	93 (52.0, 44.7–59.1)	86 (48.0, 40.8–55.3)	ns
Vomiting	173 (12.8, 11.1–14.7)	94 (54.3, 46.9–61.6)	79 (45.7, 38.4–53.1)	ns
Diarrhea	167 (12.4, 10.7–14.2)	85 (50.9, 43.4–58.4)	82 (49.1, 41.6–56.6)	ns
Dyspnea	166 (12.3, 10.6–14.2)	94 (56.6, 49.0–63.9)	72 (43.4, 36.1–51.0)	ns
Chills	165 (12.2, 10.6–14.1)	97 (58.8, 51.2–66.0)	68 (41.2, 34.0–48.8)	<0.05
Odynophagia	145 (10.7, 9.2–12.5)	98 (67.6, 59.6–74.7)	47 (32.4, 25.3–40.4)	<0.001
Myalgia	140 (10.4, 8.9–12.1)	84 (60.0, 51.7–67.7)	56 (40.0, 32.2–48.3)	<0.05
Thoracic pain	110 (8.1, 6.8–9.7)	66 (60.0, 50.6–68.7)	44 (40.0, 31.3–49.3)	<0.05
Polypnea	119 (8.8, 7.4–10.4)	63 (52.9, 44.0–61.7)	56 (47.1, 38.3–56.0)	ns
Arthralgia	102 (7.6, 6.3–9.1)	63 (61.8, 52.1–70.6)	39 (38.2, 29.4–47.9)	<0.05
Conjunctivitis	63 (4.7, 3.7–5.9)	39 (61.9, 49.5–72.9)	24 (38.1, 27.1–50.4)	ns
Anosmia	48 (3.6, 2.7–4.7)	29 (60.4, 46.3–73.0)	19 (39.6, 27.0–53.7)	ns
Cyanosis	43 (3.2, 2.4–4.3)	27 (62.8, 47.8–75.6)	16 (37.2, 24.4–52.1)	ns
Dysgeusia	39 (2.9, 2.1–3.9)	28 (71.8, 56.2–83.4)	11 (28.2, 16.5–43.8)	<0.001

Notes: HIV, human immunodeficiency virus; COPD, chronic obstructive pulmonary disease; RT-qPCR, reverse-transcription quantitative polymerase chain reaction; ND, no determinate; ns, not significant.

**Table 2:** Demographic and clinical features of the study population diagnosed by RT-qPCR related to viral load.

		Viral Load						
Variable *n* (%)	Total, *n* = 528 (%, 95% CI)	*p* Value	Low ^a^, *n* = 257 (%, 95% CI)	*p* Value	Moderate ^b^, *n* = 68 (%, 95% CI)	*p* Value	High ^c^, *n* = 203 (%, 95% CI)	*p* Value
**Gender**								
Female	241 (45.6, 41.4–50.0)	<0.05	119 (46.3, 40.3–52.4)	ns	35 (51.5, 39.8–62.9)	ns	87 (42.9, 36.2–49.7)	<0.05
Male	287 (54.4, 50.1–58.5)		138 (53.7, 47.6–59.7)		33 (48.5, 37.0–60.2)		116 (57.1, 50.3–63.7)	
**Age (years)**								
<2	77 (14.6, 11.8–17.8)	Ref	32 (12.4, 8.9–17.0)	Ref	11 (16.2, 9.3–26.7)	Ref	34 (16.7, 12.2–22.5)	Ref
2–4	92 (17.4, 14.4–20.9)	ns	47 (18.3, 14.0–23.5)	ns	9 (13.2, 7.1–23.3)	ns	36 (17.7, 13.1–23.6)	ns
5–9	135 (25.6, 22.0–29.4)	<0.001	76 (29.6, 24.3–35.4)	<0.001	11 (16.2, 9.3–26.7)	ns	48 (23.6, 18.3–29.9)	ns
10–14	127 (24.0, 20.6–27.9)	<0.001	60 (23.3, 18.6–28.9)	<0.05	21 (30.9, 21.2–42.6)	ns	46 (22.7, 17.4–28.9)	ns
15–19	97 (18.4, 15.3–21.9)	ns	42 (16.3, 12.3–21.3)	ns	16 (23.5, 15.0–34.8)	ns	39 (19.2, 14.4–25.2)	ns
**Patient care**								
Ambulatory	313 (59.3, 55.0–63.4)	<0.001	152 (59.1, 53–65)	<0.05	38 (55.9, 44.1–67.0)	ns	123 (60.6, 53.7–67.0)	<0.05
Hospitalized	215 (40.7, 36.6–45.0)		105 (40.9, 35–47)		30 (44.1, 32.9–55.9)		80 (39.4, 32.9–46.3)	
**SARS-CoV-2 infection**								
Asymptomatic	169 (32, 28.2–36.1)	ref	84 (32.7, 27.2–38.6)	ref	24 (35.3, 25.0–47.1)	ref	61 (30.0, 24.2–36.7)	ref
Mild	203 (38.4, 34.4–42.7)	ns	98 (38.1, 32.4–44.2)	ns	23 (33.8, 23.7–45.7)	ns	82 (40.4, 33.9–47.3)	ns
Moderate	134 (25.4, 21.8–29.2)	<0.05	60 (23.3, 18.6–28.9)	<0.05	19 (27.9, 18.7–39.6)	ns	55 (27.1, 21.4–33.6)	ns
Severe	22 (4.2, 2.8–6.2)	<0.001	15 (5.8, 3.6–9.4)	<0.001	2 (2.9, 0.8–10.1)	<0.001	5 (2.5, 1.0–5.6)	<0.001

Notes: ns, not significant; ^a^ Ct value 30–38 (4.2 × 10^2^–1.5 copies number/mL); ^b^ Ct value 25–29 (1.4 × 10^4^–8.5 × 10^2^ copies number/mL); ^c^ Ct value <24 (<2.8 × 10^4^ copies number/mL); ref, reference.

## Data Availability

The national data that support the findings of this study are available on https://datos.covid-19.conacyt.mx/ (accessed on 28 April 2022). The HIMFG data that support the findings of this study are available from the corresponding author (V.L.) upon reasonable request.
